# Mapping the impact, sustainability and pedagogical frameworks of international virtual knowledge exchanges in global health: Protocol for a scoping review

**DOI:** 10.12688/gatesopenres.16347.1

**Published:** 2025-05-16

**Authors:** Oisin Brady Bates, Alexandru Nicholas Grecu, Divya Iyer, Diarmuid Stokes, Walter Cullen, Joseph Gallagher

**Affiliations:** 1 University College Dublin, Dublin, Leinster, Ireland; 2 University College Dublin, Dublin, Leinster, Ireland

**Keywords:** Global Health, Public Health and Epidemiology, Health professions’ education, Healthcare professionals, Virtual Exchange, Medical Education, Literature Review, Scoping Review

## Abstract

**Rationale:**

Virtual exchanges are emerging as innovative educational tools with the potential to foster collaboration between High-Income Countries (HICs) and Low- and Middle-Income Countries (LMICs). These initiatives hold the potential to enhance intercultural competencies, promote equitable partnerships, and address resource disparities. Understanding the pedagogical underpinnings, challenges, and best practices of virtual exchanges is vital for developing scalable and sustainable integration into healthcare education.

**Research Question:**

To what extent have virtual exchanges in global health been reported in the literature to date, including their frameworks, impact and sustainability?

**Inclusion criteria:**

Studies involving a global health virtual exchange between at least one HIC and one LMIC will be included. Sources in all healthcare contexts will be included. Non-English language publications and those solely using secondary data will be excluded.

**Methods:**

The review will be conducted in line with the Joanna Briggs Institute guidance for scoping reviews (1). The following electronic databases will be searched: Medline Ovid, Embase, CINAHL & ERIC. A search of the grey literature will also be conducted. Three reviewers will independently screen the titles and abstracts and full texts for eligibility. Data extraction will be conducted independently by three reviewers. A narrative summary and tables will be presented. Key stakeholders will be consulted throughout the review.

**Discussion:**

This scoping review will provide a comprehensive understanding of virtual exchanges in global health, outlining frameworks, outcomes, content and best practices. The findings will inform the development of evidence-based models to design and sustain virtual exchanges between HIC and LMICs, enhancing their impact in global health education and practice.

**Registration:**

This protocol was registered to the Open Science Framework (OSF): DOI 10.17605/OSF.IO/MWHBP

## Key messages

1. Our scoping review will focus on virtual exchanges in global health.
2. When reporting the findings, the Preferred Reporting Items for Systematic Reviews and Meta-analyses extension for Scoping Reviews (PRISMA-ScR) will be used.
3. This study will mark the inaugural phase of a multi-part research initiative entitled “Virtual Exchanges in Primary Care (VICTORY)”.

## Ethics and consent

The study does not involve any primary data collection from human or animal subjects. As such, ethical approval is not required for this study.

### Patient and public involvement

Patients or the public were not involved in the design, or conduct, or reporting, or dissemination plans of our research.

### Transparency statement

The lead author confirms that this manuscript is an honest, accurate, and transparent account of the study being reported; no important aspects of the study have been omitted; and that any discrepancies from the study as originally planned have been explained.

## Ethics and dissemination

As no intervention or patient recruitment will be required, research ethics board approval is not applicable. We plan to disseminate our results via publication in a peer-reviewed journal or conference presentation.

## Introduction

### The need for global healthcare worker education

There is growing international recognition of the need to train a large number of additional healthcare workers in order to meet the health needs of a growing global population. It is projected that by the year 2030 there could be a shortfall of 18 million healthcare workers. ^2^ However, preparing a workforce of 40 million healthcare professionals to meet the demands of 21st-century health systems is a formidable challenge. The most significant shortages, based on need, are concentrated in South-East Asia, with a deficit of 6.9 million health workers, and Africa, with a shortfall of 4.2 million. Relative to population size, the Sub-Saharan African (SSA) region faces the most acute difficulties. ^3^

In order to address healthcare workforce shortages in low or middle-income (LMIC) settings, structural reforms in education aimed at strengthening the training capacity, relevance and adaptability of the healthcare workforce are critical in order to improve the provision of patient-centred services. Currently, 75% of the capacity to train new healthcare professionals is concentrated in upper-middle- and high-income countries. ^2^ Meanwhile, Sub-Saharan Africa (SSA), which bears 24% of the global disease burden, accounts for only 4% of the world’s health workforce—a disparity that has remained unchanged for over a decade. ^4^ To address these inequities, innovative solutions are needed, particularly in countries with the greatest unmet health needs, such as those in SSA.

E-learning modalities have been acknowledged as a potential solution to address the healthcare workforce in LMIC settings. ^5^ Indeed, the World Health Organization (WHO) has introduced new guidelines to optimize community-based health worker programs and support strategic investments in education and skill development, which include recommendations to harness information and communication technologies. ^6^

### Primary health care in Sub-Saharan Africa

There is a need to shift healthcare education systems away from narrow specialisations toward fostering locally relevant competencies that address both health and social priorities. Investment in primary healthcare systems, in particular, has the potential to confer significant benefits in resource poor settings. ^7^ The Declaration of Alma-Ata in 1978 was the first international call for PHC and was followed by the Declaration of Astana in 2018. ^8^ However, PHC is in crisis. It is underdeveloped in many countries, underfunded in others, and facing a severe workforce recruitment and retention challenge, especially in low-income countries such as those in SSA. ^9– 11^ Half the world’s population has no access to essential healthcare services. Nevertheless, 80–90% of people’s health needs across their lifetime can be provided within a primary healthcare framework. ^8^

In an editorial in 2018, WHO Director-General Tedros Adhanom Ghebreyesus and colleagues explained how the original vision of Alma-Ata has gone “largely unfulfilled”. ^12^ They highlighted that investing in PHC will support tangible improvements in health and well-being and drive progress towards achieving the health targets of the Sustainable Development Goals. Education and training to support this development are vital. The recent COVID-19 pandemic has highlighted even more the need for a strong multidisciplinary PHC workforce articulated in the Declaration of Alma-Ata and, more recently in Astana. ^13^

### Virtual knowledge exchange

The COVID-19 pandemic also highlighted the potential for virtual modalities to be utilised in education. During COVID-19 lockdown, in person education and educational exchanges were not possible, necessitating novel learning approaches. ^14, 15^ Virtual exchange (VE) programs, which connect learners from different parts of the world through technology, offer a way to address this gap. ^16, 17^ Knowledge exchange is a process in which the implicit knowledge is expressed and shared in a manner that is aimed to enhance the knowledge of exchange participants. ^18^ Virtual exchanges are knowledge exchanges that are conducted through a combination of physical and virtual platforms. ^19^ They consist of “sustained, technology-enabled, people-to-people education programmes” between “individuals or groups who are geographically separated and/or from different cultural backgrounds”. ^20^

With respect to global health, virtual exchanges have the potential to build capacity and aid in developing life-long learning for the healthcare workforce in LMICs, through partnerships between higher education institutions. ^17^ For the purposes of our study, global health virtual exchanges are defined as involving at least one High-Income Country and one Low-or Middle-Income Country.

The potential for increased accessibility to learning materials afforded by using virtual modalities also has the potential to address geographical imbalances in healthcare workforce training and distribution. However it is important to note the importance of introducing online modalities in a structured and responsible manner. ^21^ Promoting socially accountable education has been shown to successfully attract young people from underserved communities into healthcare careers. ^22^ Expanding rural and community-based education and training programs not only addresses workforce gaps but also generates employment opportunities for youth. ^23^

In low- and middle-income countries (LMICs), virtual exchanges for medical education may alleviate the burden of severe health worker shortages and deliver affordable access to high quality medical education. In combination with a e-learning approaches, international knowledge exchange offers the opportunity for healthcare students to glean novel insights within the educational process and to foster personal and professional networks, often resulting in high user satisfaction and greater long term viability of international networks. ^17, 24^

Despite the potential of global virtual exchanges to bolster healthcare workforce shortages in LMICs, there is a lack of evidence mapping their existence or evaluating their characteristics, efficacy and impact.

### Research question & aims

This scoping review will summarise the existing literature relating to global health virtual exchanges for healthcare students and professionals. For the purposes of this study, a global health virtual exchange is defined as an exchange that involves at least one LIC and one HMIC.

The primary research question is:

To what extent have virtual exchanges in global health been reported in the literature to date, including their frameworks, impact and sustainability?

The aims of the review:

• To identify and map the pedagogical frameworks underpinning virtual exchanges in global health.
• To assess the role of virtual exchanges in enhancing intercultural competencies among participants.
• To explore how virtual exchanges support sustainable and equitable partnerships between HICs and LMICs.
• To examine the challenges and best practices associated with implementing virtual exchanges in global health education.
• To examine educational outcomes and content in literature describing virtual exchanges.

**Inclusion criteria**

The Participants, Concept and Context (PCC) framework was used to develop the research question and to inform the inclusion and exclusion criteria.

### Participants

This review will include studies involving participants engaged in virtual exchanges in the context of global health. Participants may include students, educators, healthcare professionals, and researchers from both High-Income Countries (HICs) and Low- and Middle-Income Countries (LMICs). Eligible studies should explicitly describe the involvement of at least one HIC and one LMIC in the virtual exchange initiatives.

Sources relating to healthcare professionals and healthcare students will be included. Healthcare professionals are individuals who “study, diagnose, treat and prevent human illness, injury and other physical and mental impairments in accordance with the needs of the populations they serve”. This includes medical doctors, nursing and midwifery professionals, dentists and pharmacists. We will also include educators or researchers in these fields. We will exclude social care workers and social workers from this list, as they do not fall under the definition of healthcare professionals.

Only studies that involve at least one LIC and one HMIC country will be included.

### Concept

We will include competencies, curricular frameworks and learning outcomes. We will exclude sources that discuss competencies and learning outcomes without a structure or framework.

The concept focuses on virtual exchanges within the realm of global health education. This includes programs that leverage digital platforms to facilitate intercultural learning, collaborative projects, or shared health education goals between HICs and LMICs.

### Context

We will include sources in all healthcare and health professions’ education settings. This includes undergraduate, postgraduate and continuing professional education settings.

We will only include studies if they involve at least one HIC and one LMIC country.

## Methods

A scoping review was selected as the most appropriate method for this study, as it aims to explore curricular frameworks, competencies and relevant challenges and best practices relating to global health virtual exchanges, in addition to how these exchanges have been developed and their impact in establishing sustainable partnerships between HIC and LMIC countries.

The review will adhere to the Joanna Briggs Institute (JBI) methodology for scoping reviews ^1^ and will follow the Preferred Reporting Items for Systematic Reviews and Meta-Analysis extension for scoping reviews (PRISMA-ScR). ^25^ The six-step framework by Arksey and O’Malley, refined by Levac, ^26^ will guide the process: (1) formulating the research question, (2) identifying relevant studies, (3) selecting studies, (4) charting the data, (5) collating, summarizing, and reporting findings, and (6) consulting stakeholders. The protocol for this review has been registered on the OSF (DOI 10.17605/OSF.IO/MWHBP).

As this is a scoping review, the focus will be on mapping the breadth of available evidence without synthesizing findings or assessing the methodological quality of included studies.

### Search strategy

The search strategy, developed by the research team, will include studies addressing virtual exchanges in global health. Eligible works may encompass formal curricula, supplementary courses, or workshops. There will be no restrictions on study design; commentaries and expert opinions will also be included if they provide relevant insights into curriculum components, implementation of virtual exchanges, or recommendations for future development.

Searches will be conducted across MEDLINE OVID, EMBASE, CINAHL and ERIC, supplemented by manual reference list reviews and targeted grey literature searches using Google. Relevant materials will also be sought from national and international organizations, educational institutions, and professional bodies. Only studies published in English will be included, and no date restrictions will be applied. The full search strategy is detailed in Extended data, ^27^ hosted on the OSF.

### Study selection

The screening process will consist of two stages: (1) title and abstract screening, and (2) full-text review. All citations will be imported into Rayyan ( www.rayyan.ai), a Cochrane-endorsed literature screening tool. Three independent reviewers will pilot the inclusion criteria on 10% of abstracts to ensure clarity and consistency before screening the remaining studies. Disagreements will be resolved through discussion or by consulting a fourth reviewer if necessary. The inclusion criteria are detailed in Table 1. A PRISMA flow diagram will outline the selection process and a PRISMA-ScR checklist is available as supplementary material on the OSF.

**Table 1.** Eligibility criteria.

Inclusion criteria	Exclusion criteria
This review will include studies involving participants engaged in virtual exchanges in the context of global health. Participants may include students, educators, healthcare professionals, and researchers from both High-Income Countries (HICs) and Low- and Middle-Income Countries (LMICs). Eligible studies should explicitly describe the involvement of at least one HIC and one LMIC in the virtual exchange initiatives.	All papers that fall outside the inclusion criteria will be excluded. Only studies that involve at least one LIC and one HMIC country will be included.
Sources relating to healthcare professionals and healthcare students will be included.	We will exclude social care workers and social workers from this list, as they do not fall under the definition of healthcare professionals.
No restriction on study design, outcome or setting or date of publication.	Studies solely using secondary data will be excluded.
English language publications will be included.	Non-English language publications will be excluded.

### Data extraction

Two independent reviewers will extract data using a piloted data extraction tool, with disagreements resolved through discussion or consultation with the research team. Any modifications to the extraction tool during the process will be documented. Authors of included studies may be contacted for missing or additional data. Rayyan will facilitate the extraction process. A draft version of the data extraction tool is available in Extended data. ^27^

### Data analysis

Findings will be summarized narratively and presented in tables. A matrix will organize information on competencies, domains, and learning outcomes under descriptive headings. Framework analysis will guide the thematic coding of pedagogical components, competencies, and challenges/best practices associated with virtual exchanges in global health, in particular in relation to exchange impact and the establishment of sustainable partnerships between HIC and LMIC countries. The lead reviewer will draft the final report, incorporating feedback from the research team. Rigour will be maintained through an audit trail of decisions throughout the review process and through the triangulation of review findings with existing literature and ongoing stakeholder input.

### Stakeholder consultation

Stakeholder engagement, as proposed by Arksey and O’Malley, will be an integral part of this review. Health professional educators, global health experts, patient representatives, and policymakers will be consulted to ensure comprehensiveness and relevance. Stakeholders will be invited to identify additional sources and provide feedback on preliminary findings. The outcomes of these interactions will be documented and integrated into the final report.

### Study status

At the time of publication, the review is at the full-text screening stage.

## Discussion

This review will provide a comprehensive overview of existing pedagogical frameworks, competencies, and challenges related to virtual exchanges in global health. It aims to explore how virtual exchanges can support sustainable global health partnerships. It also aims to identify gaps in the literature and guide the development of a global health virtual exchange curriculum. Strengths of this review include its robust methodology, comprehensive search strategy, and stakeholder consultation to enhance relevance and applicability.

The findings will be disseminated through peer-reviewed publications and presentations at conferences. A summary of the results will be provided to key stakeholders.

## Declarations

### Ethics

Given that the scoping review methodology consists of the examination and collation of information from publicly accessible resources, ethical approval is deemed unnecessary for this study.

## Author contributions

Oisín Brady Bates: Conceptualisation, Methodology, Writing – Original Draft, Writing – Review & Editing, Project Administration

Alexandru Nicholas Grecu: Writing – Review & Editing

Divya Iyer: Writing – Review & Editing

Diarmuid Stokes: Conceptualisation, Methodology, Search Strategy, Writing – Review & Editing

Walter Cullen: Conceptualisation, Methodology, Writing – Review & Editing

 Joseph Gallagher: Conceptualisation, Methodology, Writing – Review & Editing, Project administration

## Data Availability

No underlying data are associated with this article. Open Science Framework (Dataset): Mapping the impact, sustainability and pedagogical frameworks of international virtual knowledge exchanges in global health: Protocol for a scoping review: DOI https://www.doi.org/10.17605/OSF.IO/MWHBP. ^27^ The project contains the following extended data:
1. 29.01.25 VICTORY Scoping Review PRISMA-ScR Checklist 2. 30.01.25 Virtual exchanges GH Scoping Review Protocol 3. 7.2.25 GH Virtual Exchange Review Appendix Data are available under the terms of the Creative Commons Attribution 4.0 International license (CC-BY 4.0). PRISMA SCR Checklist for “Mapping the impact, sustainability and pedagogical frameworks of international virtual knowledge exchanges in global health: Protocol for a scoping review” available at the OSF: DOI - https://www.doi.org/10.17605/OSF.IO/MWHBP. ^27^

## References

[1] Peters MDJ Marnie C Tricco AC : Updated methodological guidance for the conduct of scoping reviews. *JBI Evid. Synth.* 2020;18(10):2119–2126. 10.11124/JBIES-20-00167 33038124

[2] World Health Organization: *Working for Health and Growth: Investing in the Health Workforce—Report of the High-Level Commission on Health Employment and Economic Growth.* World Health Organization;2016.

[3] Boniol M Kunjumen T Nair TS : The global health workforce stock and distribution in 2020 and 2030: a threat to equity and ‘universal’ health coverage? *BMJ Glob. Health.* 2022;7(6):e009316. 10.1136/bmjgh-2022-009316 35760437 PMC9237893

[4] Anyangwe SCE Mtonga C : Inequities in the Global Health Workforce: The Greatest Impediment to Health in Sub-Saharan Africa. *Int. J. Environ. Res. Public Health.* 2007;4(2):93–100. 10.3390/ijerph2007040002 17617671 PMC3728573

[5] Barteit S Guzek D Jahn A : Evaluation of e-learning for medical education in low- and middle-income countries: A systematic review. *Comput. Educ.* 2020;145:103726. 10.1016/j.compedu.2019.103726 32565611 PMC7291921

[6] WHO: Global strategy on human resources for health: workforce 2030. 2016. 10.1186/s12960-024-00940-x PMC11450981 39363378

[7] WHO: Primary health care on the road to universal health coverage: 2019 monitoring report. 2019.

[8] The L : The Astana Declaration: the future of primary health care? *Lancet.* 2018;392(10156):1369. 10.1016/S0140-6736(18)32478-4 30343840

[9] Chan M : Primary health care: Now more than ever. *UN Chron.* 2012;47(2):4–7. 10.18356/cbabf986-en

[10] Flinkenflögel M Sethlare V Cubaka VK : A scoping review on family medicine in sub-Saharan Africa: practice, positioning and impact in African health care systems. *Hum. Resour. Health.* 2020;18(1):27. 10.1186/s12960-020-0455-4 32245501 PMC7126134

[11] Mash R Howe A Olayemi O : Reflections on family medicine and primary healthcare in sub-Saharan Africa. *BMJ Glob. Health.* 2018;3(Suppl 3):e000662. 10.1136/bmjgh-2017-000662 29765778 PMC5950631

[12] Ghebreyesus TA Fore H Birtanov Y : Primary health care for the 21st century, universal health coverage, and the Sustainable Development Goals. *Lancet.* 2018;392(10156):1371–1372. 10.1016/S0140-6736(18)32556-X 30343843

[13] Rasanathan K Evans TG : Primary health care, the Declaration of Astana and COVID-19. *Bull. World Health Organ.* 2020;98(11):801–808. 10.2471/BLT.20.252932 33177777 PMC7607474

[14] Keylen PLN van der Kunisch R : Asynchronous, digital teaching in times of COVID-19: a teaching example from general practice. 2020. 10.3205/zma001391 PMC7740025 33364377

[15] Hossain M : Unequal experience of COVID-induced remote schooling in four developing countries. *Int. J. Educ. Dev.* 2021;85:102446. 10.1016/j.ijedudev.2021.102446

[16] Berndt A Murray CM Kennedy K : Effectiveness of distance learning strategies for continuing professional development (CPD) for rural allied health practitioners: a systematic review. *BMC Med. Educ.* 2017;17(1):117. 10.1186/s12909-017-0949-5 28701199 PMC5506644

[17] Bridgwood B Woolley K Poppleton A : A scoping review of international virtual knowledge exchanges for healthcare professionals. *Educ. Prim. Care.* 2023;34(1):7–15. 10.1080/14739879.2022.2147025 36583515

[18] Fiedler ST Heyne T Bogner FX : COVID-19 and lockdown schooling: how digital learning environments influence semantic structures and sustainability knowledge. *Discov. Sustain.* 2021;2(1):32. 10.1007/s43621-021-00041-y 35425917 PMC8310703

[19] O’Dowd R : Virtual exchange: moving forward into the next decade. *Comput. Assist. Lang. Learn.* 2021;34(3):209–224. 10.1080/09588221.2021.1902201

[20] EVOLVE: What is Virtual Exchange? 2025. Reference Source

[21] Chimbunde P : Funding the online teaching and learning in developing countries: insights from Zimbabwe. *Educ. Technol. Res. Dev.* 2023;71(2):753–766. 10.1007/s11423-022-10163-3 36320827 PMC9615629

[22] Mahdavynia S Larijani SS Mirfakhraee H : The impact of socially accountable health professional education: Systematic review. *J. Family Med. Prim. Care.* 2022;11(12):7543–7548. 10.4103/jfmpc.jfmpc_835_22 36994039 PMC10040980

[23] WHO Guidelines Approved by the Guidelines Review Committee: *Increasing Access to Health Workers in Remote and Rural Areas Through Improved Retention: Global Policy Recommendations.* Geneva: World Health Organization Copyright © 2010, World Health Organization;2010. 23741785

[24] Ron Neville SR Wilson P Nkhoma P : The twinning of Scottish general practices and Malawian clinics: the provision of email and internet services. 2007. 10.14236/jhi.v15i1.644 17612475

[25] Tricco AC Lillie E Zarin W : PRISMA Extension for Scoping Reviews (PRISMA-ScR): Checklist and Explanation. *Ann. Intern. Med.* 2018;169(7):467–473. 10.7326/M18-0850 30178033

[26] Levac D Colquhoun H O’Brien KK : Scoping studies: advancing the methodology. *Implement. Sci.* 2010;5(1):69. 10.1186/1748-5908-5-69 20854677 PMC2954944

[27] Bates OB : Mapping the Impact, Sustainability and Pedagogical Frameworks of International Virtual Knowledge Exchanges in Global Health: Protocol for a Scoping Review.[Dataset]. *OSF.* 2025 February 7. 10.17605/OSF.IO/MWHBP PMC12084517 40386084

